# PCR-SSCP Variation of IGF1 and PIT1 Genes and Their Association with Estimated Breeding Values of Growth Traits in Makooei Sheep

**DOI:** 10.1155/2013/272346

**Published:** 2013-12-09

**Authors:** Masoud Negahdary, Abbas Hajihosseinlo, Marziyeh Ajdary

**Affiliations:** ^1^Yazd Cardiovascular Research Center, Shahid Sadoughi University of Medical Sciences, Yazd, Iran; ^2^Department of Animal Science, Faculty of Agriculture, Urmia University, Urmia, Iran; ^3^Young Researchers and Elites Club, Khorasgan Branch, Islamic Azad University, Isfahan, Iran

## Abstract

Molecular biology techniques genetic improvement by facilitating identification, mapping and analysis of polymorphism of genes by encoding proteins that act on metabolic pathways involved in economically interesting traits. This use of genetic markers can aid identification of those animals with the highest breeding values in sheep. On the basis of sheep genome mapping, information was examined on the ovine IGF1 and PIT1 genes as a possible genetic marker for growth traits in sheep. The current study was designed to estimate the frequencies of putative IGF-1 and PIT-1 genes SNPs and investigate associations with calculated EBVs of growth traits in Makooei sheep. PCR-SSCP analysis of the exon1 of IGF-I gene and include a part of intron2, exon3 and a part of intron3 and PIT-1 gene revealed the following banding patterns; three (AA, AG, GG) and four AA (p1), AB (p2), CC (p3), CD (p4), banding patterns respectively. Results from this study demonstrated higher performance of AA animals in BW and GBW, and AG animal in WW and W6 that may be related to the role of IGF-1 at the pre-puberty and puberty stages. Also higher performance of p3 animals in W9, YW and GSN, and p1 animal in GNY may be related to the PIT-1 role in post-puberty.

## 1. Introduction

The Makooei sheep are one of the Iranian fat-tailed, medium-size breeds. They are distributed in the mountainous areas of the country, especially in West Azerbaijan province. Also, they are found in Turkey and called White Karaman. They are valuable primarily for meat and also for their wool and milk. The wool produced is coarse and usually used for carpet weaving [1]. The main objective of the application of molecular biology techniques to animal genetic improvement programs currently consists in identifying, mapping, and analyzing polymorphisms of the genes involved in the main metabolic pathways that are related to animal growth and distribution of nutrients to the different tissues [2]. Recently, investigators and breeders focus on marker-assisted selection (MAS) and genome analysis. MAS may increase annual rate of genetic gain in livestock by 15 to 30% without increasing the risk involved in breeding schemes [3]. In the livestock industry, growth traits that determine economic value of livestock are always of primary concern during breeding [4]. The most significant growth traits for birth cohort studies in genetics are gestation, litter size, sex, and environmental variables [4]. In farm animals, promising candidate genes for many traits are in the growth hormone (GH) axis. The GH gene pathway contains various interdependent genes, such as GH, insulin-like growth factor1 (IGF1), pituitary specific transcription factor1 (PIT1), growth hormone releasing hormone (GHRH), somatostatin growth hormone releasing hormone receptor (GHRHR), growth hormone receptor (GHR), and others [5]. For growth traits, GH, GHR, insulin-like growth factor I (IGF-I), leptin (LEP), caprine-pituitary-specific transcription factor-1 (POU1F1), caprine myostatin (MSTN), and bone morphogenetic protein (BMP) genes are necessary for bone formation, birth weight, weaning weight, body condition, and muscle growth [6]. IGF1 is a mediator of many biological effects: it increases absorption of glucose, stimulates myogenesis, inhibits apoptosis, participates in the activation of cell cycle genes, increases the synthesis of lipids, stimulates the production of progesterone in granular cells, and intervenes in the synthesis of DNA, protein, RNA, and functions in cell proliferation [7]. The predicted sequence of amino acid oIGF-I peptide differs from the human, bovine, and porcine IGF-Is at a single amino acid (at position 66, alanine is substituted for proline) and differs from rat and mouse IGF-Is at positions 4 and 5, respectively. Ovine IGF-I amino-terminal peptides are 1 amino acid longer than other mammalian IGFs due to the presence of an extra amino acid (glutamine) that is present at the proposed boundary of exon1 and exon2 [8]. IGF-1 is one of the most potent natural activators of the AKT signaling pathway, a stimulator of cell growth and multiplication and a potent inhibitor of programmed cell death. The transcription factor pituitary-1 (Pit-1, official nomenclature now POU1F1) plays a major role in pituitary development and hormone expression. It has been shown to be a positive regulator of the growth hormone, prolactin, and the thyrotropin subunit (TSH*β*) in the mammalian pituitary [9]. POU1F1 is a member of the POU-domain gene family. The POU-domain is composed of high-affinity DNA bonds, the POU-specific domain (POU-SD), and a POU-homeodomain (POU-HD) [10]. Mutations of the POU1F1 gene led to a deficiency or an absence of GH, PRL, and TSH-*β* and can result in stunted growth. Mammalian growth and development require steady expression of the POU1F1 gene [10]. The ovine POU1F1 gene has a high level of conservation with its bovine, human, and rat counterparts showing 98.2%, 91.2%, and 86.2% of similarity at coding levels, respectively [10]. Such findings suggest that variation in IGF-1 and PIT-1 genes in domestic animals may be important contributors to growth rate. However, studies on associations of IGF-1 and PIT-1 Polymorphisms with growth traits are mainly carried out in cattle but examination of associations between SNPs and growth traits in these genes has not been reported in sheep. Therefore, the objective of this study was to determine associations of genotypes with estimated breeding values of growth traits in Makooei sheep.

## 2. Materials and Methods

### 2.1. Collection of Sheep Blood Samples and Extraction of Genomic DNA Extraction

The Makooei breed of sheep were examined in this study; they are fat-tailed sheep with medium body size, white in color with black spots on face and feet. They are farmed in the East and West Azerbaijan provinces of Iran for meat and wool [11]. Blood samples were collected into a 5 mL EDTA vacutainer tube and transferred to the laboratory within 2 hours for DNA extraction. Total DNA extractions were made with a modified salting out method [12] from whole fresh blood. Quality and quantity of extracted DNA were measured on 0.8% agarose gel prepared in 0.5x TBE buffer (45 Mm Tris base, 45 Mm boric acid, and 1 mM EDTA pH 8.0), visualized with ethidium bromide (1.0 *μ*gml^−1^), and photographed under UV light. Estimates of variance components and genetic parameters for birth weight (BW), weaning weight (WW), 6-month weight (6MW), 9-month weight (9MW), and yearling weight (YW) from single-trait analyses are presented in Table 1.

### 2.2. PIT1 Genotyping

The PIT1 gene was genotyped by PCR-SSCP (polymerase chain reaction-single strand conformation polymorphism). A 295 bp fragment was amplified from a part of intron2, exon3 and a part of intron3. The primers used for the procedure were those designed by [10]: PIT-1-uP: 5′-GAGGGATAATTACAAATGGTCC-3 and PIT-1-down: 5′-TGTTAACAGCTGTGGGACACAC-3′. PCR contained 25–50 ng genomic DNA, 10 pmoL of each primer, 2 *μ*L 10x PCR buffer, 1.5 mM MgCl2, 200 Mm dNTP, and 1 unit Taq-polymerase, in a total volume of 20 *μ*L. DNA amplifications were performed using a Master cycler (Eppendorf, Germany) programmed for a preliminary step of 15 min at 95°C, followed by 34 cycles of 45 s at 94°C then 1 min at 58°C, followed by 45 s at 72°C, and finally an extension of 5 min at 72°C.

### 2.3. IGF-1 Genotyping

As primer pair, IGF-1-up (5′-ATTACAG CTGCCTGCCCCTT-3′) and IGF-1-down (5′-CACATCTGCTAATACACCTTACCCG-3′) targeting a fragment of 265 bp were employed in DNA amplifications as described by [13]. PCR contained 25–50 ng genomic DNA, 10 pmoL of each primer, 2 *μ*L 10x PCR buffer, 1.5 mM MgCl2, 200 Mm dNTP, and 1 unit Taq-polymerase, in a volume of 20 *μ*L. DNA amplifications were done using a Master cycler programmed for a preliminary step of 2 min at 95°C, followed by 31 cycles of 45 s at 94°C, 30 s at 58°C, and 30 s at 72°C, with a final extension of 3 min at 72°C.

### 2.4. Single Strand Confirmation Polymorphism (SSCP)

Two pairs of oligonucleotide primers were designed and a standard PCR protocol was used to amplify two fragments.

SSCP analysis: several factors were tested to optimize the methodology: amount of PCR product (4–15 *μ*L), dilution in denaturing solution (20–85%), denaturing solution (A: 95% of formamide, 10 mM NaOH, 0.05% xylene-cyanol, and 0.05% bromophenol blue; B: same as A plus 20 mM of EDTA), acrylamide concentration (6–14%), 6 percentage for PIT1, 7 percentage for IGF-1, percentage of crosslinking (1.5 to 5%), presence (10%) or absence of glycerol, voltage (100–50 V), running time (2–12 h), and running temperature (4, 6, 10, and 15°C). Each PCR reaction was diluted in denaturing solution, denatured at 95°C for 5 min, chilled on ice, and resolved on a nondenaturing polyacrylamide gel. The electrophoresis was carried out in a vertical unit (Payapajoohesh VEU-7350, 160 × 140 × 0.75 mm) in 1x TBE buffer. The gels were stained with silver.

### 2.5. Data Collection

Data were collected at the Makooei Sheep Breeding Station at Makoo (36°, 35′S and 48°, 22′E) in West Azerbaijan province. Animals are kept on natural pasture during spring, summer, and autumn seasons. Range conditions are poor during the winter months and, therefore, animals are kept indoors during the winter. In general, the flock is managed under a semimigratory system.

### 2.6. Breeding Value Estimation and Association Analysis

The analysis was conducted using restricted maximum likelihood (REML) using a derivative-free (DF) algorithm procedure [14]. Data on growth traits were retrieved from the national Sheep Recording System (SRS). The following fixed effects model was used to calculate BV (breeding value) with DFRIMEL software [14]:

(1) Yijklmno=μ+YRi+SXj+BTk+ADl+ANm+Mn+Eijklmno

with *σ*_*am*_ ≠ 0, where *μ* is overall mean of population; *Y*_*ijk*…_… is each observation; YR_*i*_ is fixed effect of year *i*; SX_*j*_ is fixed effect of sex *j*; BT_*k*_ is fixed effect of birth type *k*; AD_*l*_ is fixed effect of age of dam *l*; AN_*m*_ is additive genetic effect of animal *m*; *M*_*n*_ is maternal genetic effect; *E*_*ijk*…_ is residual effect of an observation *ijk*….

The estimated parameters according to model were: phenotypic variance (*σ*_*p*_^2^), direct additive genetic variance (*σ*_*a*_^2^), maternal genetic variance (*σ*_*m*_^2^), residual variance (*σ*_*e*_^2^), direct heritability (*h*_*a*_^2^), (*σ*_*a*_^2^/*σ*_*p*_^2^), and maternal heritability (*h*_*m*_^2^), (*σ*_*m*_^2^/*σ*_p_^2^).

SAS software was used to calculate least squares means and for multiple comparisons among the different genotypes in “Makooei” sheep. Least square analysis using the general linear model (GLM) procedure was done to identify the fixed effects on a model that were to be included in the model and then the model was made using the fixed effects (sex: 2 classes; type of birth: 2 classes; IGF-1: 3 classes; PIT-1: 4 classes), according to the following statistical model:

(2) $$\begin{array}{c} Y_{ijklm}=\mu +G_{i}+P_{d}+S_{j}+ls_{k}\;+G_{i}\times \text{L}{\text{S}}_{k}+e_{ijklm}, \end{array}$$

where *Y*_*ijklm*_ is growth traits, *μ* is the overall mean, *G*_*i*_ is the fixed effect of the *i*th genotype for IGF-1, Pd is the fixed effect of the dth genotype for PIT-1, *S*_*j*_ is the fixed effect of sex (*j* = 1, 2), *l*_*sk*_ is the fixed effect of litter size (*k* = 1, 2), Gi.LS is the interaction between genotype for IGF-1 and litter size, and *e*_*ijklm*_ is the random residual error.

### 2.7. Statistical Analysis

All data were stored in SAS for operating system of Microsoft Co. (version 19). Group comparisons were done using the analysis of variance (ANOVA) test. Significant differences between them were assessed by Student's *t*-test. All data were expressed as mean ± standard error of mean (SEM). *P* values less than 0.05 were considered significant.

## 3. Results

### 3.1. Genomic Screening

PCR-SSCP analysis of the 5′ flank region (exon1) of the ovine IGF-I gene and (a part of intron2, exon3 and a part of intron3) PIT-1 gene revealed the following banding patterns: three (AA, AG, GG) and four (AA (p1), AB (p2), CC (p3), CD (p4)) banding patterns, respectively. Chi-square analysis has shown that the genotypic frequency for the PIT1 (*P* < 0.05) was not in the Hardy-Weinberg equilibrium, but the frequency for IGF-1 (*P* > 0.05) was in the Hardy-Weinberg equilibrium.

### 3.2. Genetic Parameter Estimation

Estimates of variance and covariance components were based on animal models using the restricted maximum likelihood (REML) approach in a derivate-free (DF) algorithm [14]. The direct heritability estimate of the studied traits was fixed while the maternal heritability estimates varied between 0.07 and 0.22. Safari et al. have reviewed genetic parameter estimates for sheep growth traits and the heritability means for growth traits ranged from 0.15 to 0.41. Genetic evaluation for body weights prior to 6 months of growth traits needs to adopt a model that considers genetic effects, both direct and maternal genetic ones. Therefore, the current study was designed to estimate the effect of SSCP patterns on weight estimated matenal breeding values for body weights prior to 6-months of growth traits. Low additive genetic and high residual variances in BW, WW, 6MW, 9MW, and YW could be explained by the harsh environmental conditions of the range that coincided with these ages. The high genetic and phenotypic correlation between weaning weight with 6-month weight, 9-month weight and yearling weight indicates that a breeding ram may be selected at an earlier age for the PIIT-1 and IGF-1 genes (Table 4).

### 3.3. Effect of IGF-I SSCP Variants on the Growth Traits

Effects of the tested SNPs on the EBVs for growth traits in Makooei sheep are presented in Table 2. The table demonstrates that we observed a significant effect of this polymorphism on 6MW yield (*P* < 0.05). The genotype AG has the highest additive estimated breeding value for the trait pre-six month weight (SW). The effect of the IGF-I gene was significant (*P* < 0.01) for average daily gain from birth to weaning (GBW), birth weight (BW), weaning weight (WW), six-month weight (SW), and average daily gain from six months to nine months (GSN). In the tested Makooei sheep population, mean body weight of the genotype AG at 6MW (31.12 kg) was about 26.9 kg, and this was 7.13 higher than those of AA (28.43 kg) and GG (23.99 kg) SSCP patterns, respectively. Also mean of wool weights of genotype AG at yearling weight (0.517) was about 0.031 kg and 0.172 higher than that of AA (0.486) and GG (0.345) SSCP patterns, respectively. Higher performance of AA animals in BW and GBW, also AG animal in WW and *W*_6_ (prepuberty ages) may be related to the IGF-1 role in prepuberty ages. We suggest that decrease weight of animals has been due to change of genotype.

### 3.4. Effect of PIT-1 SSCP Variants on the Growth Traits

The results showed that the PIT-1 genotypes were associated with estimated breeding values of growth traits (Table 3). A significant effect of this polymorphism was observed on 9MW yield (*P* < 0.01). The p4 genotype had the highest additive estimated breeding value for nine-month weight (9MW). The p3 genotype had the highest weight for all traits except *W*_6_ and GWS. The effect of the PIT-1 gene was significant (*P* < 0.01) for nine-month weight (9W), from age six months to nine months (GSN), and from nine months to yearling weight (GNY). In the tested Makooei sheep, population mean body weights of p3 genotype at 9MW (30.98 kg) were about 2.25 kg and 2.80 kg and 3.29 kg higher than those of the SSCP patterns p4 (28.43 kg) and p2 (28.18) and p1 (27.69), respectively. Higher performance of p3 animals in *W*_9_ and GSN, also p1 animal in GNY (postpuberty ages), may be related to role of PIT-1 in postpuberty.

## 4. Discussion

Most traits with economic interest in animal production showed continuous variation. However, their underlying genetic nature is very complex. It is possible to identify the chromosome regions containing genes affecting these traits because of the current availability of neutral polymorphisms scattered throughout the genome [15]. Growth is a composite of complex developments that are influenced by genetic, nutritional, and environmental factors. Preweaning (BW, WW, GBW) and Postweaning (6MW, 9MW, YW, GWS, GSY) growth traits have an important impact on the profitability in the sheep industry. Therefore, breeding for optimal body weight (BW) and larger gains is a major consideration in sheep breeding programs.

The SSCP analysis of those genes, supposed to have an association with production traits, could be a valuable alternative approach to establish the allelic variants that are useful as markers to aid in selection. Many several studies have been done on the association of IGF-1, PIT-1 genes polymorphisms and other traits in animals. Up to now, there were very few reports about IGF-1, PIT-1 genes SNPs and their relationship with growth and development traits of animals, especially for sheep. Genetic variations of the POU1F1 gene on growth traits of Nanyang Cattle showed that body weight at 12 months was higher in the BB individuals than in the AA individuals (*P* < 0.05) [16]. The statistical analysis showed that polymorphism of the IGF-I gene had a significant association (*P* < 0.05) with birth weight (BW), body weight at 6 months (*W*_6_) and at 12 months (*W*_12_) in Nanjing Huang goats. The goats with genotype CC had significantly higher BW, *W*_6_, *W*_12_ than those with genotype GC and had significantly higher *W*_12_ than those with genotype GG. The body weight at 12 months was higher in the BB individuals than in the AA individuals (*P* < 0.05) [17].

Until now, a few polymorphisms of IGF-1 and PIT-1 genes have been detected in small ruminants and sheep have been studied much less. Very little information is currently available to compare different Iranian sheep breeds. To date, this was the (first) study that attempted to detect allele variation in the ovine IGF-1, PIT-1 genes and its association with breeding value of growth traits in Iranian sheep breeds. The previous breeding programs in most research centers of Iran were based on only phenotypic characters. The current study confirmed the importance of molecular studies beside the morphological data in detecting genetic variation among individuals in selecting diverse parents to construct a new population. Thus, adding sheep varieties, expanding samples, and doing further correlation studies are still needed to accumulate quantitative molecular genetics data in studying the relationship between the IGF-1, PIT-1 genes and growth traits in sheep.

In conclusion, these results indicate that this polymorphism contributed to variation in the traits analyzed and reinforced the possibility of using these polymorphisms in molecular marker-assisted selection and breeding programs.

## Figures and Tables

**Table 1:** Parameters estimated of analyzed traits.

Traits	σ_a_^2^	σ_m_^2^	σ_e_^2^	σ_p_^2^	h^2^	m^2^
BW	0.045	0.015	0.14	0.22	0.2	0.07
WW	1.6	0.96	4.90	8.32	0.2	0.12
6MW	2.59	2.81	6.48	12.95	0.2	0.22
9MW	2.35	2.04	6.8	11.75	0.2	0.17
YW	3.29	1.74	10.38	16.35	0.2	0.11

**Table:** (a)

IGF-1	Weight estimated maternal breeding values (means ± SE, kg)		
	BW	W_3_	W_6_
AA	0.006 ± 0.02	0.595 ± 0.14	1.02 ± 0.27^ab^
AG	0.005 ± 0.03	0.873 ± 0.17	1.45 ± 0.34^a^
GG	0.026 ± 0.04	0.294 ± 0.24	0.158 ± 0.48^b^

*P* value	0.3^ns^	2.32^ns^	2.93*

**Table:** (b)

IGF-1	Weight estimated breeding values (means ± SE, kg)				
	BW	W_3_	W_6_	W_9_	W_12_
AA	−0.063 ± 0.04	1.18 ± 0.28	1.45 ± 0.47^ab^	0.965 ± 0.25	0.304 ± 0.30
AG	−0.083 ± 0.05	1.75 ± 0.35	2.4 ± 0.59^a^	0.945 ± 0.31	0.285 ± 0.37
GG	−0.24 ± 0.07	0.588 ± 0.51	0.155 ± 0.83^b^	0.527 ± 0.44	0.337 ± 0.53

*P* value	2.5^ns^	2.33^ns^	2.95*	0.48^ns^	0.0^ns^

^ns^not significant; *significant; ^a^AG; ^b^GG; ^ab^AA.

**Table:** (a)

PIT-1	Weight estimated breeding values (means ± SE, kg)				
	BW	W_3_	W_6_	W_9_	W_12_
P1	−0.066 ± 0.03	1.017 ± 0.23	0.963 ± 0.281	0.167 ± 0.20^a^	0.225 ± 0.595
P2	−0.082 ± 0.06	0.708 ± 0.44	0.438 ± 0.321	0.651 ± 0.39^ab^	0.338 ± 0.670
P3	−0.098 ± 0.04	1.61 ± 0. 27	1.40 ± 0.218	0.890 ± 0.24^b^	0.250 ± 0.654
P4	−0.27 ± 0.11	1.36 ± 0.64	2.65 ± 1.08	1.54 ± 0.57^ab^	0.128 ± 0.69

*P* value	1.40^ns^	1.92^ns^	1.19^ns^	3.66*	0.16^ns^

**Table:** (b)

PIT-1	Weight estimated maternal breeding values (means ± SE, kg)		
	BW	W_3_	W_6_
P1	−0.005 ± 0.01	0.510 ± 0.23	0.452 ± 0.22
P2	−0.006 ± 0.03	0.347 ± 0.21	0.726 ± 0.42
P3	−0.015 ± 0.02	0.826 ± 0. 13	0.832 ± 0.26
P4	−0.051 ± 0.05	0.666 ± 0.32	1.51 ± 0.62

*P* value	0.66^ns^	1.30^ns^	2.21^ns^

*P* value: significant level; ^ns^not significant; *significant; ^a^AG; ^b^GG; ^ab^AA.

**Table 4:** Correlation between traits in Iranian “Makui” sheep breed resulted from SAS analysis.

Trait 1	Trait 2	r_p12_	r_a12_
WW	6MW	0.77	0.75
WW	9MW	0.61	0.56
WW	YW	0.54	0.32

6MW	9MW	0.75	0.93
6MW	YW	0.59	0.80
9MW	YW	0.73	0.87

r_p12_: phenotypic correlation between trait 1 and trait 2; r_a12_: direct additive genetic correlation between trait 1 and trait 2.
